# List of Reviewers

**DOI:** 10.1093/jrr/rraf076

**Published:** 2025-11-26

We would like to express our greatest appreciation to our reviewers. *Journal of Radiation Research* Vol. 66 (2025) benefited from the comments and insights from the following 248 reviewers. We sincerely thank them for their time devoted and effort which contributed to the improvement of the journal. (Reviewers who reviewed three or more manuscripts are listed in bold font.)

Raizulnasuha Ab Rashid

Tadesse Abate

**Kota Abe**

Takanori Abe

Yu Abe

Suryakanta Acharya

Rihito Aizawa

Keiichi Akahane

Naofumi Akata

Tetsuo Akimoto

Yuichi Akino

B. Almayahi

Selvakumar Anbalagan

Ken Ando

Koichi Ando

Gabor Andocs

**Takuro Ariga**

Hidetaka Arimura

Hirofumi Asakura

Mikhail Balonov

Tomoki Bo

Kamil Çam

Chuanhui Cao

Weishan Chang

Yixing Chen

**James Chow**

Hikmettin Demir

Yusuke Demizu

Ibrahima Diallo

Nan Ding

Takeshi Ebara

Yasuo Ejima

Krzysztof Fornalski

Toshioh Fujibuchi

Nariaki Fujimoto

Yukio Fujita

Winky Wing Ki Fung

Hiraku Fuse

Maksym Gusyev

Akihiro Haga

Yasushi Hamamoto

Kanya Hamasaki

Wei Han

Takashi Hanada

**Hideyuki Harada**

Hiroshi Harada

Takayuki Hashimoto

**Masaharu Hata**

Yoshiomi Hatayama

Yoshihide Hattori

Naoki Hayashi

Masaharu Hazawa

Joichi Heianna

Shinya Hiraoka

Hideaki Hirashima

Takero Hirata

Ryoichi Hirayama

Nobuyuki Hirohashi

Masaharu Hoshi

Naonori Hu

Hiroshi Igaki

Noriko Ii

Daisuke Iizuka

Nobuki Imano

Ken Inoue

Hiraku Iramina

Junya Ishikawa

Tetsuo Ishikawa

Hiromichi Ishiyama

**Fumiaki Isohashi**

Satoshi Itasaka

Hitoshi Ito

Alexander Ivannikov

Hiromitsu Iwata

Keiichi Jingu

**Noriyuki Kadoya**

Michiaki Kai

Atsushi Kaida

Tomohiro Kajikawa

Hidemi Kamezawa

Yuko Kaneyasu

Hiroyuki Katoh

Norio Katoh

Shinji Kawabata

Hidemasa Kawamura

Mariko Kawamura

Kohei Kawata

Masahiro Kenjo

**Tomoki Kimura**

Noriko Kishi

Satoshi Kito

Yusuke Koba

Yoshiro Kobayashi

Seiji Kodama

Akito Koganezawa

Masaoki Kohzaki

Takahumi Komiyama

**Takashi Kondo**

Teruaki Konishi

Chutima Kranrod

Hiromi Kudo

Nobuki Kudo

Hiroaki Kumada

Satoshi Kumagai

Kyo Kume

Osamu Kurihara

Chie Kurokawa

Takeaki Kusada

Jian Jian Li

Katsuya Maebayashi

Munetoshi Maeda

Hirokazu Makishima

Koji Masui

Hiroaki Matsubara

Yoshihisa Matsumoto

Yoshitaka Matsumoto

Yukinori Matsuo

Kazumasa Minami

Takamasa Mitsuyoshi

Yuki Miyabe

**Naoki Miyamoto**

Yuya Miyasaka

Yusaku Miyata

Masashi Mizumoto

**Shinichiro Mori**

Akinori Morita

Takahito Moriwaki

Naoya Murakami

Yuji Murakami

Kazutoshi Murata

Yusa Muroya

Masaki Nagane

Tetsuo Nakajima

Takahiro Nakamoto

Katsumasa Nakamura

**Mitsuhiro Nakamura**

Satoaki Nakamura

Satoshi Nakamura

**Hisashi Nakano**

Ikuno Nishibuchi

Kentaro Nishioka

Tetsuo Nonaka

**Toshiyuki Ogata**

Mitsuaki Ojima

Kohei Okada

Hiroyuki Okamoto

Masahiko Okamoto

Noriyuki Okonogi

Kae Okuma

Yasutaka Omori

Kaoru Ono

Takashi Ono

**Tomohiro Ono**

Seiichi Ota

Kensuke Otsuka

Natsuo Oya

Oona Rainio

**Masahide Saito**

Tetsuo Saito

Makoto Sakai

Katsuyuki Sakanaka

Dosatsu Sakata

Ritsu Sakata

**Vasanthan Sakthivel**

Takayuki Sakurai

Tomonori Sakurai

Tetsuya Sanada

Yu Sanada

Maira Santos

Keisuke Sasai

Megumi Sasatani

Yoshiaki Sato

Yuta Sekino

Shintaro Shiba

Yuta Shibamoto

Hidetoshi Shimizu

Tsutomu Shimura

Makoto Shinoto

Katsuyuki Shirai

Kenshiro Shiraishi

Masanori Someya

**Valeriy Stepanenko**

Iori Sumida

Shizuyo Sutou

Gen Suzuki

Keiji Suzuki

Masatoshi Suzuki

Motofumi Suzuki

Akira Tachibana

Masataka Taga

Senzo Taguchi

Masashi Takada

Takeo Takahashi

Yasuyuki Takahashi

Yutaka Takahashi

**Shigeyuki Takamatsu**

Ryoko Takasawa

Masaaki Takashina

Shino Takeda

Hideki Takegawa

Hideyuki Takei

Yasutaka Takei

Satoshi Takeno

Tsuguhide Takeshima

**Yuki Tamakuma**

Satoshi Tanabe

**Kenichi Tanaka**

Osamu Tanaka

Hiroshi Tauchi

Hirofumi Tazoe

Atsuro Terahara

Takafumi Toita

Koichi Tokuue

Yuki Tominaga

**Masanori Tomita**

Chisato Tonoiso

Toshiyuki Toshito

Norio Tsujimura

Kayoko Tsujino

Masato Tsuneda

Haruka Uezono

Rei Umezawa

Megumi Uto

Satoru Utsunomiya

Maria Varnava

Takahiro Wada

**Yuki Wada**

Nobuhide Wakai

Yushi Wakisaka

Bing Wang

Kohshin Washiyama

Tsubasa Watanabe

Sonja Wegener

Kentaro Yamamoto

Chikako Yamauchi

**Hideya Yamazaki**

Hiroshi Yasuda

**Koichi Yasuda**

Hironobu Yasui

**Keisuke Yasui**

Sumi Yokoyama

Kengo Yoshida

Yuya Yoshimoto

Michio Yoshimura

Shinji Yoshinaga

**Hironori Yoshino**

Yuki Yoshino

Yasuo Yoshioka

